# Orchestrating epigenetics: a comprehensive review of the methyltransferase SETD6

**DOI:** 10.1038/s12276-025-01423-2

**Published:** 2025-03-18

**Authors:** Anand Chopra, Michal Feldman, Dan Levy

**Affiliations:** 1 The Shraga Segal Department of Microbiology, Immunology and Genetics, Ben-Gurion University of the Negev, Be’er-Sheva, Israel; 2 National Institute for Biotechnology in the Negev, Ben-Gurion University of the Negev, Be’er-Sheva, Israel

**Keywords:** Methylation, Biochemistry

## Abstract

Transcription is regulated by an intricate and extensive network of regulatory factors that impinge upon target genes. This process involves crosstalk between a plethora of factors that include chromatin structure, transcription factors and posttranslational modifications (PTMs). Among PTMs, lysine methylation has emerged as a key transcription regulatory PTM that occurs on histone and non-histone proteins, and several enzymatic regulators of lysine methylation are attractive targets for disease intervention. SET domain-containing protein 6 (SETD6) is a mono-methyltransferase that promotes the methylation of multiple transcription factors and other proteins involved in the regulation of gene expression programs. Many of these SETD6 substrates, such as the canonical SETD6 substrate RELA, are linked to cellular pathways that are highly relevant to human health and disease. Furthermore, SETD6 regulates numerous cancerous phenotypes and guards cancer cells from apoptosis. In the past 15 years, our knowledge of SETD6 substrate methylation and the biological roles of this enzyme has grown immensely. Here we provide a comprehensive overview of SETD6 that will enhance our understanding of this enzyme’s role in chromatin and in selective transcriptional control, the contextual biological roles of this enzyme, and the molecular mechanisms and pathways in which SETD6 is involved, and we highlight the major trends in the SETD6 field.

## The methyltransferase SETD6: an epigenetic enzyme involved in human diseases

The chemical modification of proteins, such as lysine methylation, influences their role in the cell. This review focuses on an epigenetic enzyme called SETD6 that adds small chemical methyl groups to certain proteins. Over the past 15 years, research on SETD6 has shown that it plays an important role in human health and disease. The interactions of SETD6 with other proteins, particularly its ability to chemically modify them, have been shown to influence the individual functions of these proteins. This influence affects cells at the epigenetic level—affecting gene regulation—as well as cell behavior. Whether SETD6 promotes or suppresses cancerous properties depends on the type of cancer. Therefore, research focusing on the role of SETD6 in the cell is crucial to understand whether SETD6 could be a promising target for therapeutic intervention.

.

## Introduction

Lysine methylation is a dynamic posttranslational modification (PTM) that regulates protein function and biological processes. After the preliminary discovery of methylated lysine on a bacterial flagellar protein in 1959 (ref. ^1^), the involvement of histone lysine methylation in transcription was established in the 1960s (ref. ^2^). However, it was nearly four decades later that the first mammalian histone lysine methyltransferase (KMT) was discovered^3^. Now, over 25 histone methyltransferases are known to confer site- and state-specific histone lysine methylation, and these modifications are key regulators of fundamental biological processes and diseases^4,5^. The methylation of non-histone proteins is also closely connected to cellular signaling and the regulation of cancer pathways^6,7^. So far, the lysine methylome consists of over 10,000 unique modification sites^8^. The majority of the methylome consists of non-histone proteins, and only a fraction of these modification sites have been mapped to specific KMTs with functional outcomes.

The catalytic activity of canonical KMTs is attributed to Su(var)3-9–enhancer of zeste–trithorax (SET) domains. SET domains require a cofactor, *S*-adenosyl-l-methionine (SAM), as a methyl donor to add methyl groups (–CH_3_) to the ε-amino group of lysine residues in the form of mono-, di- or tri-methylation^6^. Approximately 55 human proteins contain SET domains, and among these, approximately 25 distinct histone KMTs have been identified^4,7^. The enzymatic activity of numerous histone KMTs is now known to regulate biological processes through non-histone methylation. Unlike other KMTs, SET domain-containing protein 6 (SETD6) was first discovered as a non-histone KMT^9^. Most physiological SETD6 substrates are non-histone proteins that are involved in transcriptional regulation, and the role of SETD6 in numerous biological processes and signaling pathways has become increasingly evident. SETD6 has an expansive network of in vitro protein substrates that will play a role in directing the future of the SETD6 field. Here, we provide a comprehensive overview of all aspects inherent to SETD6, such as its structure and regulation, enzymatic activity, control over transcriptional programs, as well as its role in cell biology and disease. In addition, we synthesize knowledge from the past 15 years of SETD6 research and provide research hypotheses that will pave the way for future endeavors in the SETD6 field.

## SETD6 structure and function

Phylogenetic analysis of human protein methyltransferases revealed that SETD6 was most closely related to SETD3 and SETD4 (ref. ^10^). Previous formal classifications have described these three methyltransferases as belonging to a group with non-histone methylation activity (class VII), which includes the rubisco large- and small-subunit methyltransferases^11,12^.

**Fig. 1:**
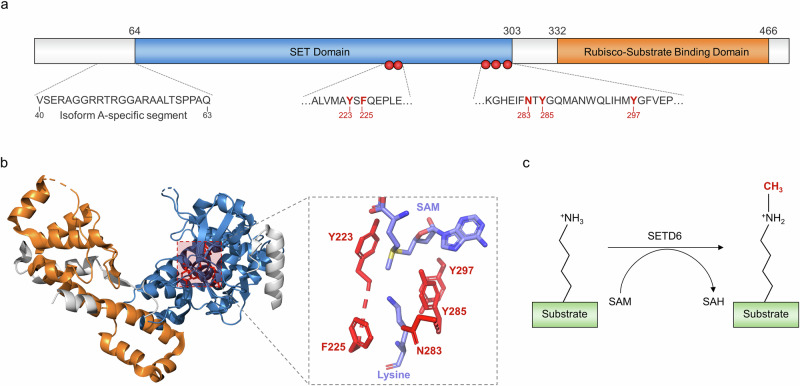
Domain organization, structure, and function of SETD6. **a** SETD6 is composed of two main functional domains: the SET domain (blue) and the Rubisco-substrate binding domain (orange). The residues forming the active site are depicted by red spheres. Isoform A has a 23-residue segment that is not present in isoform B. **b** The crystal structure of SETD6 (PDB: 3QXY) depicts the three-dimensional arrangement of the functional domains and the active site (same coloring as in **a**). The enlargement depicts the active site residues (red), *S*-adenosyl methionine (SAM) cofactor and substrate lysine; the latter two are colored on the basis of atoms (magenta, carbon; blue, nitrogen; red, oxygen; yellow, sulfur). **c** SETD6 uses the SAM cofactor to catalyze mono-methylation of substrate lysine residues.

Among plant methyltransferases, KMTs within this classification have been shown to have a truncated or interrupted SET domain and lack a diverse array of additional domains (readers, zinc fingers, DNA binding domains and so on) that are present in other methyltransferase classes^12^. The *SETD6* gene is located within chromosome 16 (location 16q21) and encodes a protein that contains two main domains: the catalytic SET domain (residues 64–302) and the Rubisco-substrate binding domain (residues 332–465) (Fig. 1a). Furthermore, SETD6 may exist as either a longer 473-residue (isoform A) or shorter 449-residue (isoform B) splice variant, and the longer variant has a 23-residue insertion directly before the SET domain (Fig. 1a)^13^. Within the catalytic SET domain, five residues (Y223, F225, N283, Y285 and Y297) form a hydrophobic active site that engages the aliphatic side chain of a substrate lysine residue (Fig. 1a,b). In addition, the Y285 residue directly contacts the *S*-adenosyl methionine cofactor, and the mutation of this residue to alanine abolishes SETD6 activity. With respect to product specificity, SETD6 preferentially catalyzes mono-methylation of its canonical substrate, and this phenomenon holds true for the vast majority of SETD6 substrates (Fig. 1c)^9,13^. The Rubisco substrate-binding domain is an essential component of SETD6 enzymatic activity, as deletion of this region abolishes lysine methylation activity while retaining the ability of the SET domain to bind substrate proteins^14^. However, the precise mechanism underlying this process is not clear.

## SETD6 expression and localization

**Fig. 2:**
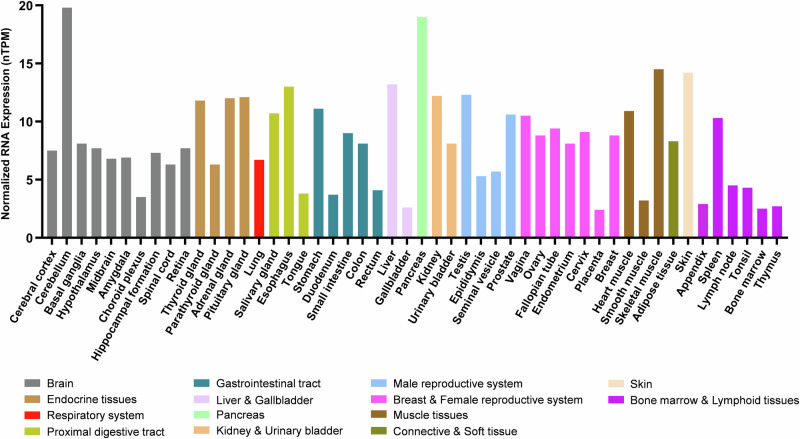
Expression of *SETD6* in various tissues. RNA expression of *SETD6* in different human tissues (colored by common functional features). The *y* axis indicates normalized expression levels (nTPM). The data in the graph are from The Human Protein Atlas ‘Consensus Dataset’ for RNA expression (https://www.proteinatlas.org/).

From a comparative biology standpoint, orthologs of human *SETD6* have been identified in two fish species: the mangrove rivulus (*Kryptolebias marmoratus*) and the common carp (*Cyprinus carpio*)^15,16^. The expression of *CcSetd6* was examined in adult common carp and was shown to be higher in female tissues than in male tissues (gonads, heart, kidney and brain), with the exception of the liver^15^. With respect to human biology, publicly available RNA expression data from the Human Protein Atlas (https://www.proteinatlas.org/) demonstrate that RNA expression of *SETD6* occurs in a wide range of tissues, with no specificity for a given tissue type (Fig. 2)^17^. Although many KMTs are expressed in this manner, tissue-specific KMT expression has been observed^18^.

**Fig. 3:**
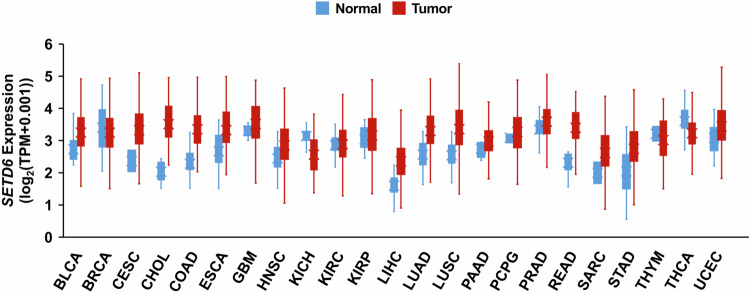
Differential expression of *SETD6* in multiple cancers. *SETD6* expression data in TCGA tumors (red) and normal tumor-adjacent tissues (blue) were obtained from UCSC Xena^19^ (https://xenabrowser.net/). *SETD6* expression data from both tissue types were taken from the ‘RSEM TPM’ expression dataset. BLCA, bladder urothelial carcinoma; BRCA, breast invasive carcinoma; CESC, cervical and endocervical cancer; CHOL, cholangiocarcinoma; COAD, colon adenocarcinoma; ESCA, esophageal carcinoma; GBM, glioblastoma multiforme; HNSC, head and neck squamous cell carcinoma; KICH, kidney chromophobe; KIRC, kidney clear cell carcinoma; KIRP, kidney papillary cell carcinoma; LIHC, liver hepatocellular carcinoma; LUAD, lung adenocarcinoma; LUSC, lung squamous cell carcinoma; PAAD, pancreatic adenocarcinoma; PCPG, pheochromocytoma and paraganglioma; PRAD, prostate adenocarcinoma; READ, rectum adenocarcinoma; SARC, sarcoma; STAD, stomach adenocarcinoma; THYM, thymoma; THCA, thyroid carcinoma; UCEC, uterine corpus endometrioid carcinoma. These results shown here are based upon data generated by the TCGA Research Network (https://www.cancer.gov/tcga).

Furthermore, *SETD6* expression is dysregulated in several types of cancer. Analysis of publicly available gene expression data from The Cancer Genome Atlas (TCGA; https://www.cancer.gov/tcga) with UCSC Xena revealed differential expression of *SETD6* between various TCGA tumors and tumor-adjacent normal tissues^19^ (Fig. 3). Among these, SETD6 protein expression has been confirmed to be strikingly upregulated in lung adenocarcinoma tumors^20^. Furthermore, SETD6 has been shown to be upregulated at the mRNA and protein levels in transformed bladder epithelial cells (SVHUC1) and several bladder cancer cell lines (T24, RT4 and UMUC3), as well as in oral squamous cell carcinoma (OSCC)^21,22^. Other analyses have revealed that *SETD6* expression is elevated in samples from patients with glioma and that high SETD6 expression is correlated with poor patient prognosis^23^. The expression of endogenous SETD6 mRNA and protein has been observed in a wide variety of cell lines that can be used as models for studying the role of SETD6 in these tumor types (Table 1).

**Table 1:** Cell lines demonstrating endogenous SETD6 mRNA or protein expression.

Description	Cell line	Evidence
Human embryonic kidney	HEK293T	WB^9,23,24^
Human glioma	U251	WB^23^
Human osteosarcoma	U2OS	qPCR^9^ WB^9,55^
Human monocyte from acute monocytic leukemia patient	THP-1	qPCR^9^
Human monocyte-derived macrophages	n/a	qPCR^29^
Human prostate cancer	DU145	qPCR^27^ WB^27^
Human breast epithelial cells	MCF10A	WB^76^
Human breast cancer	MDA-MBA-231	qPCR^30,86^ WB^24,25,30,59,60,76^
	MCF7	qPCR^86^ WB^76^
	T47D	qPCR^86^ WB^76^
	BT549	WB^76^
Human lung cancer	A549	WB^20,24^
Human cervical cancer	HeLa	WB^53,91^
Human cervical intraepithelial neoplasm	CIN612	WB^53^
Normal human gastric epithelial cells	GES-1	WB^31^
Human gastric cancer	HGC-27	WB^31^
	SGC-7901	WB^31^
Transformed bladder epithelial cells	SVHUC1	qPCR^21^ WB^21^
Bladder cancer	T24	qPCR^21^ WB^21^
	RT4	qPCR^21^ WB^21^
	UMUC3	qPCR^21^ WB^21^
Human erythromyeloblastoid leukemia	K562	Mass spectrometry^30,98^
Mouse embryonic stem cells	E14	qPCR^55^ WB^55^
Primary mouse bone-marrow-derived macrophage	mBMDM	qPCR^9^
Mouse fibroblast	3T3	WB^9^
Mouse podocytes	n/a	WB^88^

qPCR, quantitative polymerase chain reaction; WB, western blot.

With respect to its subcellular localization, fluorescence microscopy has shown that SETD6 is concentrated in the nucleus^14^. Indeed, SETD6 localizes to chromatin, where it elicits the methylation of several chromatin-associated proteins, which will be discussed later in this Review^9,23–25^. Localization to the nucleus and chromatin is most likely driven by the N-terminus, given that loss of the C-terminal half does not impact SETD6 localization patterns^14^. In addition, biochemical fractionation has demonstrated that SETD6 is present in the cytosol^14,24^. Indeed, publicly available subcellular localization data from the Human Protein Atlas (https://www.proteinatlas.org/) show that SETD6 localizes to the nucleoplasm in human embryonic kidney (HEK293T), prostate cancer (PC-3) and osteosarcoma (U2OS) cells; in addition, SETD6 is present in the cytosol of the latter two cell lines. Relevantly, cellular protein substrates of SETD6 demonstrate localization to both nuclear and extranuclear compartments. Notably, the factors that dictate SETD6 localization, such as the presence of nuclear localization and export signals, have yet to be identified.

## Regulation of SETD6 function

SETD6 is regulated at multiple levels, and we provide an overview of the genomic, transcriptional, post-transcriptional and post-translational regulation of SETD6 function.

### Genetic mutations

In cattle, a single-nucleotide polymorphism (SNP) may occur within the bovine *SETD6* gene that results in the A360E mutation residing within the Rubisco-substrate binding domain^26^. Compared with wild-type bovine SETD6, this mutation increases SETD6 methyltransferase activity. Interestingly, the presence of a glutamine residue in this region of SETD6 is conserved from chickens to humans. Besides this, a whole-exome sequencing study of familial type X colorectal cancer identified a truncating mutation in the *SETD6* gene that leads to loss of the C-terminal half of the SETD6 protein^14^. Although loss of the Rubisco-substrate binding domain does not affect subcellular localization or binding to SETD6 substrates, the enzymatic activity of this truncated mutant is defective and impairs the function of wild-type SETD6 by competing for its substrates^14^.

### Factors regulating SETD6 expression

Little is known about the regulation of *SETD6* expression, but differences in *SETD6* expression between different tissues and cancers indicate that its expression is meaningful and tightly orchestrated (Figs. 2 and 3). The promoter region of *SETD6* is in an open chromatin state, as it is enriched in active marks of transcription, H3K27ac and H3K4me3, in prostate cells^27^. Furthermore, 48 transcription factors are predicted to bind the *SETD6* promotor region^28^. Among these, E2F1 was enriched at the *SETD6* promotor, specifically in prostate cells, which enhances *SETD6* transcription^27^. As discussed later, SETD6 participates in a positive feedback loop with E2F1 to promote *SETD6* transcription. Furthermore, SETD6 mRNA levels are downregulated by the cytokines IFN-γ and IL-4 in human monocyte-derived macrophages^29^. Moreover, oxidative stress, induced by treatment with H_2_O_2_ or arsenite, has been shown to downregulate SETD6 mRNA and protein levels^30^. Finally, SETD6 expression is post-transcriptionally downregulated by the miR-411 microRNA^31^.

### Post-translational regulation of SETD6

With respect to PTMs, SETD6 auto-methylation affects oligomerization and enzymatic activity^32^. SETD6 exists as a monomer, as oligomers (dimer and trimer) and as higher-molecular-weight aggregates^32^. Automethylation occurs on the K39, K179 and K372 residues. The auto-methylation activity stabilizes the oligomeric states of SETD6 while destabilizing higher-molecular-weight aggregates. Interestingly, the auto-methylation of K179 is required for the formation of the trimeric state. This control over self-oligomerization is directly tied to SETD6 activity, as methylation of the two major auto-methylation sites, K39 and K179, enhances SETD6 enzymatic activity^32^. In addition to lysine methylation, SETD6 harbors five ubiquitination sites and four phosphorylation sites, whereby three phosphorylated residues (S118, S120 and S138) are located within the SET domain and four ubiquitinated residues (K327, K385, K394 and K441) are within or near the Rubisco-substrate binding domain^33–35^. The K39 auto-methylation site is also known to be ubiquitinated; thus, there may be a functional interplay between ubiquitination and SETD6 oligomerization. Overall, the functional consequences of these PTMs are unknown, but modifications in these domains may impact SETD6 enzymatic activity.

## The enzyme–substrate network of SETD6

SETD6 has an extensive enzyme–substrate network that encompasses primarily in vitro protein substrates. However, an increasing number of these substrates have been validated in a cellular context, many of which are involved in chromatin and transcriptional regulation, which has significant implications in cell biology. Below, we review the substrates of SETD6 and the downstream consequences of methylation (summarized in Table 2). We distinguish between in vitro substrates and those that have been validated in a cellular context; substrate methylation that has been shown to occur both in a SETD6-dependent manner and in cells is defined as a physiological substrate.

**Table 2:** Summary of physiological SETD6 substrates and downstream effects of SETD6-dependent methylation.

Protein (UniProt ID)	Residue	Summary of consequences
RELA (Q04206)	K310	Mono-methylation represses transcriptional activity of RELA under basal conditions. Recruits binding of GLP ankyrin repeat domains^9^.
TWIST1 (Q15672)	K33	Mono-methylation increases the occupancy of TWIST1 at the *LINC-PINT* locus, leading to the enrichment of EZH2 and H3K27me3 at this region. Downregulates LINC-PINT expression^23^.
E2F1 (Q01094)	K117	Mono-methylation increases E2F1 occupancy and activity at the SETD6 promotor^27^.
BRD4 (O60885)	K99	Mono-methylation of BRD4 blocks the selective recruitment of E2F1 to genes involved in mRNA translation^25^.
H2AZ (P0C0S5)	K7	Further elucidation is required. Global levels of K7me1 increase during mouse embryonic stem cell differentiation, whereas levels near the *Fgf5* TSS decrease^55^.
PLK1 (P53350)	K209 and K413	Methylation of PLK1 suppresses its catalytic activity and slows mitotic progression^59^.
PAK4 (O96013)	K473	Methylation of PAK4 activates β-catenin transcriptional activity and confers paxillin mislocalization, thereby negatively regulating cell adhesion, migration and invasion^24,60^.
MRPS23 (Q9Y3D9)	K108	Methylation of MRPS23-K108 promotes protein stability, which counteracts the effect of MRPS23-R21 methylation^76^.
Histone H4 (P62805)	K12	Unknown^57^.

### Physiological substrates

#### Methylation of chromatin-bound transcription factors

RELA, a subunit of the NF-κB complex, was the first discovered in vitro and physiological substrate of SETD6^9^. Specifically, SETD6 catalyzes mono-methylation of the RELA lysine-310 residue (RELA-K310me1) in vitro and in several tissue culture cell lines, such as HEK293T and U2OS cells. Fractionation experiments performed with these two cell lines revealed that RELA-K310me1 localizes exclusively to chromatin under basal conditions. Furthermore, the levels of RELA-K310me1 at the promoters of RELA target genes (*IL8*, *IL1A*, *MYC* and *CCND1*) decreased upon depletion of SETD6 in U2OS and acute monocytic leukemia cells (THP-1), and it was also shown that SETD6 represses RELA transactivation activity in U2OS, THP-1 and primary mouse bone-marrow-derived macrophage (mBMDM) cells. Thus, mono-methylation of RELA by SETD6 is a repressive modification occurring at chromatin.

**Fig. 4:**
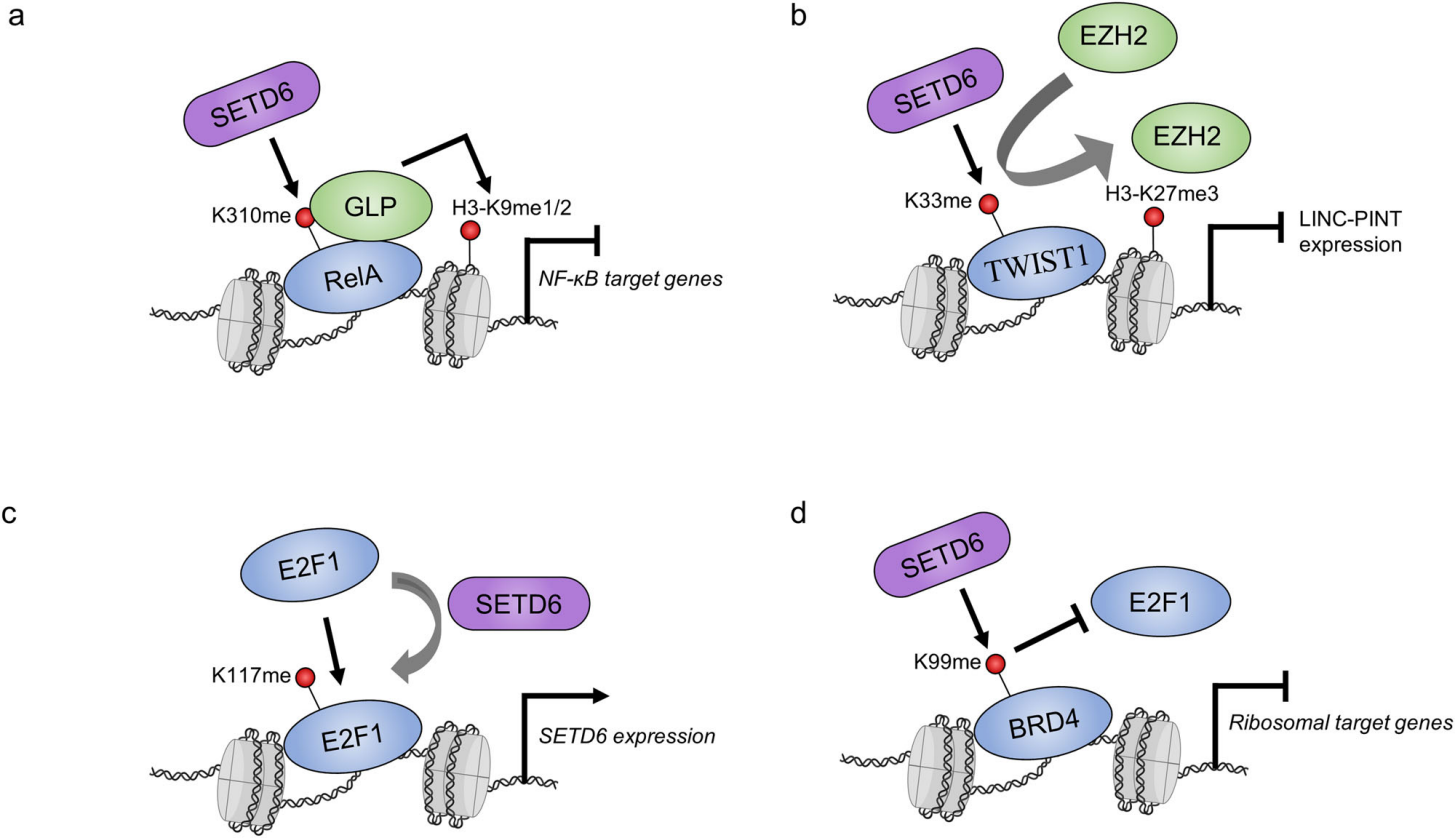
SETD6 methylation of chromatin-bound transcription factors. **a** SETD6 methylation of RELA recruits GLP, which deposits repressive H3-K9me1/2 marks and causes transcriptional repression of NF-κB target genes. **b** Methylation of TWIST1 on the *LINC-PINT* locus promotes the recruitment of EZH2 and the accumulation of H3-K27me3, which causes transcriptional repression. **c** SETD6 methylation of E2F1 promotes its binding to the *SETD6* promotor. **d** BRD4 methylation blocks E2F1 recruitment to ribosomal target genes. Purple, SETD6; blue, SETD6 substrates; green, other proteins; red, methylation sites. The nucleosome icon was created in BioRender.com.

The physiological consequences of RELA-K310 mono-methylation are repressed RELA-driven cell proliferation, as well as repressed inflammatory cytokine gene expression and secretion. Mechanistically, mono-methylation of RELA at K310 represses RELA activity by recruiting a heterochromatin-associated protein known as G9a-like protein (GLP)^9^ (Fig. 4a). Methyl-reader domains, known as ankyrin repeat domains, within GLP bind to RELA-K310me1. This represses RELA function and restrains the inflammatory response under basal conditions. However, this negative signaling is switched off upon NF-κB activation; phosphorylation at the adjacent serine-311 (S311ph) residue by atypical protein kinase C (PKCζ) inhibits GLP binding and is followed by a reduction in the repressive H3-K9me2 mark at chromatin. The localization of RELA-K310me1 is directly controlled by PKCζ phosphorylation of RELA at serine-311. Although S311ph occurs on the same molecule as K310me1 to block GLP binding, it was also shown that S311ph can block SETD6 from performing the initial methylation event in vitro^13^. RELA is also subject to acetylation at K310 by p300/CBP and mono-methylation at the K314 and K315 residues by Set7/9 (refs. ^36,37^). There is functional crosstalk between these two modifications, and future studies should elucidate how K310 methylation by SETD6 affects adjacent modifications in this PTM hub^38^.

TWIST1 is a basic helix‒loop‒helix-type transcription factor, and it is known as a master regulator of epithelial-to-mesenchymal transition (EMT) in various cancers, whereby TWIST1 promotes increased metastatic and invasive phenotypes^39^. In particular, TWIST1 is highly expressed in human glioblastoma^40^. Notably, Admoni-Elisha and colleagues found SETD6 to catalyze monomethylation of the TWIST1 lysine-33 residue (TWIST1-K33me1) in vitro as well as in HEK293T and a human glioma cell line (U251)^23^. Specifically, both the interaction and the methylated form of TWIST1 are present on chromatin.

SETD6 mRNA levels in patients with glioma are positively correlated with those of several transcription factors that are known to be involved in EMT, such as TWIST1. Furthermore, RNA sequencing analysis of wild-type and SETD6-knockout glioma cells revealed that among downregulated genes there was a significant enrichment for those involved in EMT^23^. Notably, EMT phenotypes promoted by TWIST1 (loss of cell adhesion and gain of migration) are SETD6 dependent. RNA sequencing and ChIP assays have revealed the mechanism by which SETD6 regulates TWIST1 function: methylated TWIST1 physically binds to the long intergenic p53-induced transcript (*LINC-PINT*) locus and attenuates the expression of this long noncoding RNA. LINC-PINT is regulated by p53 and is known to inhibit tumor cell invasion. Further, it is downregulated in several cancers, including GBM^41–43^. Notably, LINC-PINT suppresses EMT in GBM and other cancer models through multiple mechanisms^42,44^.

Overall, SETD6 methylation of TWIST1-K33 leads to the inhibition of *LINC-PINT* expression, which drives EMT phenotypes such as increased cell invasion and migration. This methylation represses LINC-PINT by recruiting EZH2 and its enzymatic product, the repressive H3-K27me3 mark, at the *LINC-PINT* locus (Fig. 4b). The exact mechanism underlying this selective recruitment is unknown; however, EZH2 is the catalytic subunit of polycomb repressive complex 2 (PRC2), within which several complex members harbor methyl-binding domains^45^. It would be interesting to test whether any of these domains within the PRC2 complex recognizes methylated TWIST1. Future studies should explore the molecular interactions that connect TWIST1 methylation and the recruitment of EZH2 at the *LINC-PINT* locus.

E2F transcription factors (E2Fs) represent an eight-member family (E2F1–E2F8) and are involved in a wide variety of biological processes, particularly cell proliferation, differentiation and apoptosis^46^. E2F1 was the first member of this family to be discovered and is a downstream target of the retinoblastoma protein, an important tumor suppressor and a master cell cycle regulator^47,48^. While investigating the high expression level of SETD6 in prostate cancer, Kublanovsky and colleagues discovered that E2F1 occupies the *SETD6* promoter and upregulates SETD6 mRNA expression^27^. E2F1 occupancy and transcriptional activity at the *SETD6* promoter are augmented by mono-methylation of E2F1 at the lysine-117 residue (E2F1-K117me1). In this manner, E2F1-K117 methylation facilitates a positive feedback mechanism, whereby the upregulation of SETD6 by E2F1 increases E2F1-K117me1 levels and further enhances SETD6 mRNA expression (Fig. 4c). Interestingly, the K117 residue, in addition to other adjacent lysine residues, is acetylated and regulates E2F1 at multiple levels^49–51^. Future studies should investigate the functional crosstalk between E2F1-K117 acetylation and methylation. Further, additional studies are needed to understand the biological importance of this positive-feedback loop in prostate cancer as well as other cancer cell models that demonstrate a positive correlation between *E2F1* and *SETD6* gene expression levels.

BRD4 is a transcription factor in the bromodomain and extraterminal (BET) protein family^52^. Members of this family possess two bromodomains that act as readers of lysine acetylation, particularly on chromatin, and thus are involved in the recruitment of transcription factors to target genes. SETD6 mono-methylates BRD4 at the lysine-99 residue (BRD4-K99me1), which resides in bromodomain 1 (BD1), and the methylated form of BRD4 is located on chromatin^25^. Specific to conditions of hypomethylation (for example, SETD6 knockout or BRD4-K99R expression), RNA sequencing experiments have revealed the upregulation of genes involved in mRNA translation and ribosomal RNA processing pathways. In alignment with this, conditions of hypomethylation lead to increased global translation, which further supports the finding that BRD4-K99me1 inhibits translation. Although the methylation site resides in the BRD4-BD1 region, methylation does not affect the overall recognition of acetylated histone H4 at chromatin but rather blocks the recruitment of E2F1 to specific genes involved in mRNA translation^25^ (Fig. 4d). Conversely, BRD4-K99 methylation by SETD6 was later found to enhance the association of BRD4 with the human papillomavirus (HPV) E2 protein^53^. Overall, SETD6 and BRD4 associate with the HPV-31 enhancer region within the long control region, and BRD4-K99 methylation enhances E2-dependent HPV transcriptional activation. Thus, BRD4-K99me1 directs selective transcription by regulating BRD4 interaction partners. BRD4 phosphorylation at the adjacent Y97/98 residues regulates its stabilization, chromatin binding, binding to BET inhibitors and association with STAT3 (ref. ^54^). Studying whether there is functional crosstalk between these phosphorylation and methylation sites may enable us to further uncover how methylation affects BRD4 association with other transcriptional regulators.

#### SETD6 regulates histone methylation marks

In vitro, SETD6 methylates the N-terminus of H2AZ primarily at the lysine-7 residue, and weaker methylation occurs at lysine-4 (ref. ^55^). Only cellular levels of H2AZ-K7me1 have been shown to be dependent on SETD6. In addition, global levels of both H2AZ-K7me1 and the doubly methylated H2AZ-K4me1-K7me1 have been shown to increase upon the induction of mouse embryonic stem cell differentiation. It is thought that SETD6 maintains stem cell self-renewal through the repression of select target genes via the deposition of H2AZ methylation^55^. In addition to histone H2A isoforms, SETD6 was found to methylate histone H4 in vitro and in prostate cancer cells. Methylation of H4-K12, in vitro and in prostate cancer cells, has been shown to be facilitated by HEMK2, a bifunctional glutamine and lysine methyltransferase^56^. However, at the peptide and protein levels, SETD6 is estimated to be over 1,000 times more active than HEMK2 at catalyzing the methylation of H4-K12 (ref. ^57^). In vitro methylation of histone H4 by SETD6 was previously reported on multiple lysine residues^58^. Besides the potent in vitro methylation of H4-K12 by SETD6, weaker methylations of adjacent H4-K5 and H4-K8 residues have been shown to occur at the protein level^57^. Finally, H4-K12 mono-methylation was strongly attenuated upon SETD6 KO in the DU145 prostate cancer cell line. In the context of HEMK2-dependent experimental design, H4-K12me1 has been linked to the regulation of androgen-independent proliferation of prostate tumor cells^56^. Thus, future studies are needed to determine whether SETD6 also participates in this process by regulating this methylation site.

#### Methylation of protein kinases regulates mitotic progression and cell adhesion

SETD6 regulates multiple protein kinases through its methyltransferase activity. Thus far, SETD6 is known to directly catalyze lysine methylation of Polo-like kinase 1 (PLK1) and p21-activated kinase 4 (PAK4), and these modifications have significant effects on cell biology^24,59,60^.

**Fig. 5:**
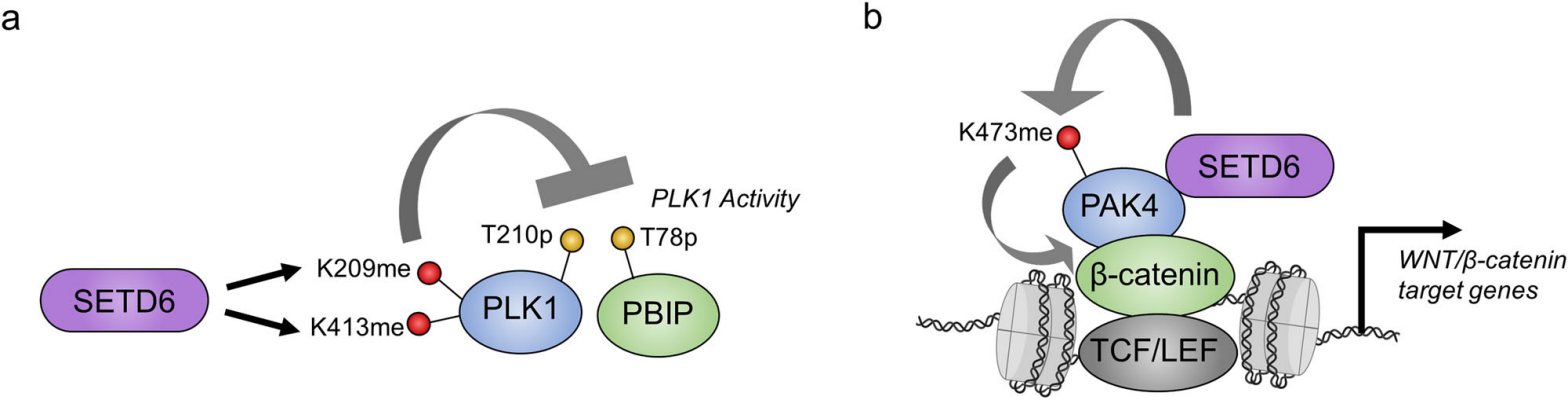
SETD6 methylation of protein kinases. **a** SETD6 methylation of PLK1 attenuates its protein kinase activity. **b** PAK4 methylation on chromatin increases its association with β-catenin, which promotes the transcription of Wnt/β-catenin target genes. The color coding is the same as in Fig. 4.

PLK1 is a master regulator of mitosis through its phosphorylation of multiple protein substrates. Importantly, PLK1-catalyzed phosphorylation of numerous substrates links this mitotic kinase to the regulation of multiple essential stages of the cell cycle and mitosis, such as M phase entry and exit, cytokinesis, sister chromatid cohesion, and other essential phases^61,62^. Multiple PTMs confer regulatory control over PLK1; for example, phosphorylation of the threonine-210 residue (T210) by Aurora A and B kinases, which reside in the PLK1 activation loop, is an essential modification for PLK1 activity^63,64^. Specifically, SETD6 directly mono-methylates PLK1 on two distinct residues, K209 and K413, one of which is directly adjacent to the T210 phosphorylation site^59^. Notably, SETD6 knockout has been shown to increase the mitotic progression of HeLa cells, which also corresponded to a shorter duration of time in different stages of mitosis, thereby increasing cell proliferation. PLK1 kinase activity, measured by the phosphorylation of PLK1-T210 and PBIBP1-T78 (an established substrate of PLK1), was also increased in SETD6-knockout cells. Correspondingly, the catalytic activity of the methylation-deficient PLK1 double-mutant was augmented, which resulted in accelerated mitosis. Thus, SETD6 methylation activity is a negative regulator of PLK1 catalytic activity and restrains cell proliferation by slowing mitotic progression^59^ (Fig. 5a). The exact mechanism by which PLK1 methylation attenuates its kinase activity is unclear, but the methylation of PLK1 at K209 may directly impact the phosphorylation of the adjacent T210 residue and methylation at K413 may impact the function of the W414 residue in substrate recognition^59,65^.

Further, SETD6 is a negative regulator of the Wnt/β-catenin signaling pathway through the methylation of p21-activated kinase 4 (PAK4) at the K473 residue^24,60^ (Fig. 5b). This substrate is a serine/threonine protein kinase that regulates numerous cellular functions, including cell adhesion, invasion and proliferation^66^. In particular, PAK4 phosphorylates β-catenin at serine-675, which attenuates the ubiquitination and subsequent proteasomal degradation of β-catenin, thereby activating the Wnt/β-catenin signaling pathway^67^. Interestingly, the methylation of PAK4 by SETD6 occurs on chromatin^24^. SETD6 directly promotes the interaction between PAK4 and β-catenin on chromatin through the formation of a complex with these two proteins, whereby SETD6 is a critical component for the activation of β-catenin transcriptional activity^24^. In breast cancer cells, PAK4 methylation by SETD6 significantly attenuated cell adhesion by causing the mislocalization of paxillin, a PAK4 substrate, thereby promoting the disassembly of focal adhesions^60^. Furthermore, the loss of cell adhesion caused by PAK4 methylation is correlated with defective migration and proliferation of breast cancer cells. Because this methylation site is adjacent to the S474ph autophosphorylation site^68^, it would be interesting to assess whether there is functional crosstalk between these two modifications.

#### Mitochondrial ribosomal protein methylation by SETD6 regulates breast cancer metastasis

The Warburg effect is a hallmark of cancer, whereby cancer cells shift from oxidative phosphorylation to aerobic glycolysis as the primary means of ATP production^69,70^. The inhibition of oxidative phosphorylation and the subsequent increase in reactive oxygen species are known to promote the metastatic potential of cancer^71,72^. Mitochondrial ribosomal protein S23 (MRPS23) is elevated in diverse human cancers, promotes several malignant properties in glioma and contributes to the proliferation of hepatocellular carcinoma and breast cancer^73–75^. In breast cancer, the methylation of MRPS23 by both SETD6 and PRMT7 regulates these cellular processes and controls metastasis^76^. In vitro, SETD6 was found to catalyze di-methylation of MRPS23 at the lysine-108 residue, which differs from the mono-methyl product specificity that has been observed for all other SETD6 substrates^76^. The methylations catalyzed by PRMT7 and SETD6 have opposing effects on MRPS23 protein stability; K108 methylation counteracts the accelerated degradation of the MRPS23 protein that is induced by PRMT7 methylation of arginine-21. It has been proposed that this competition between SETD6 and PRMT7 functions to maintain low levels of MRPS23 (ref. ^76^).

### In vitro protein substrates

WD repeat domain 5 (WDR5) is a core member of the SET1/MLL complex and is responsible for complex assembly by playing a scaffolding role, among its other functions^77,78^. Yao and colleagues reported that endogenous WDR5 is mono-methylated at lysines 207 and 325, and that the methylation of these two residues is important for WDR5-driven cell proliferation and migration as well as proper maintenance of global H3-K4me3 levels^79^. Furthermore, the methylation of these two sites was specific to SETD6 enzymatic activity in vitro, whereas SET7/9, SET8 and SMYD3 were unable to methylate WDR5. Further studies should elucidate whether SETD6 catalyzes WDR5 methylation in a physiological setting.

The application of high-throughput protein arrays has revealed that SETD6 has an expansive in vitro substrate network^80^. A total of 206 in vitro protein substrates were identified using two independent detection methods and include the PLK1 and PAK4 methylation sites mentioned earlier. Several other proteins from this screen were validated using traditional in vitro methylation methods, including transcription elongation factor A protein 1 (TCEA1), the ribosomal protein RPS27L and the splicing factors DNAJC8 and SRSF2 (ref. ^80^). Separately (and relevant to histone methylation), SETD6 was found to confer in vitro methylation of canonical histones, linker histone H1 and a reader of histone methylation known as ING2 (ref. ^58^).

### Substrate specificity

**Fig. 6:**
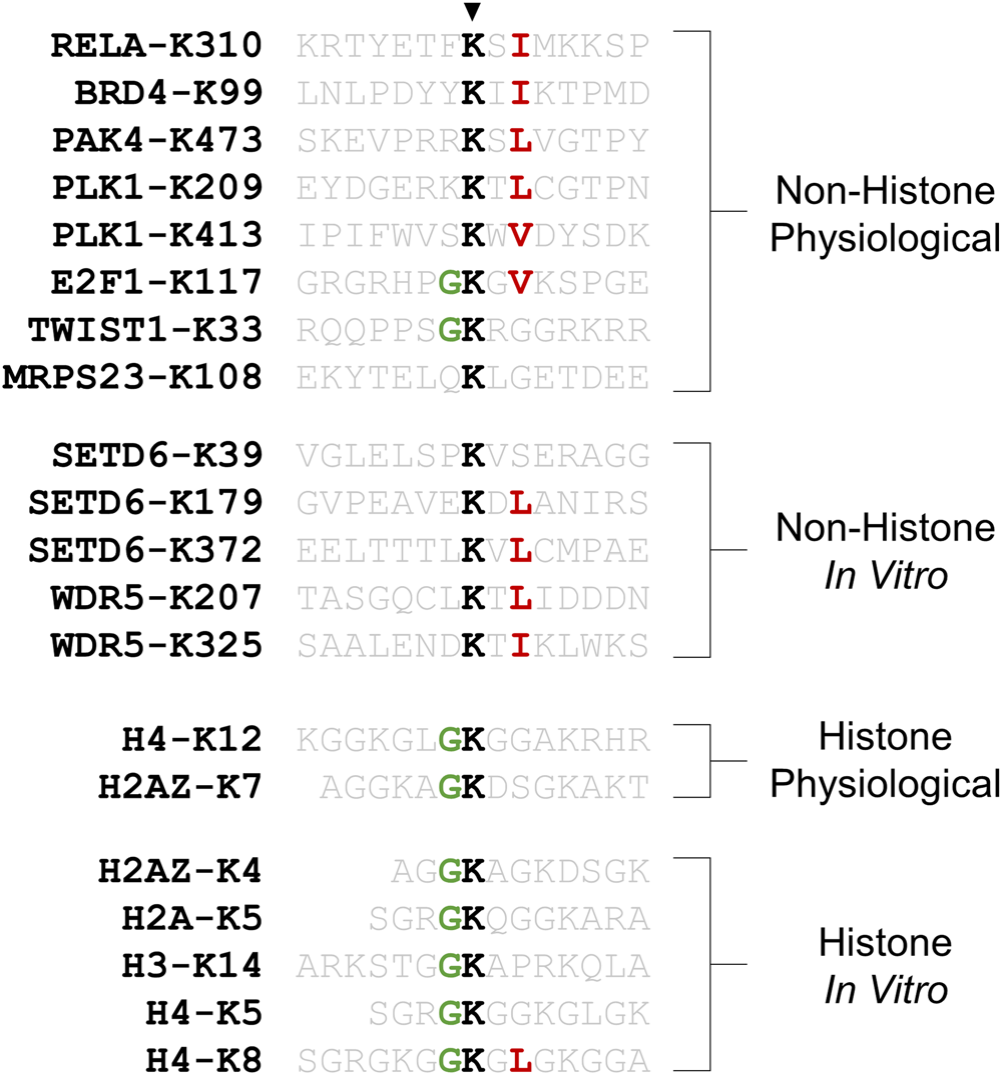
Sequence commonalities among lysine-defined SETD6 substrates. Substrates are classified into four categories based on whether they are a histone or non-histone substrate and whether methylation is known to occur in a physiological environment or is only known to occur in an in vitro context.

SETD6 has a broad range of in vitro and physiological protein substrates, the latter of the two with defined target lysine residues. Research on the recognition of the RELA peptide and protein substrates by SETD6 has revealed that regions outside of the RELA target site influence enzyme–substrate affinity and catalytic efficiency^81^. However, it is generally accepted that methylation and demethylation are directed by preferred substrate recognition motifs. The substrate recognition motifs of numerous KMTs and lysine demethylases (KDMs) have been deciphered using systematic peptide libraries^82–85^, but such methodologies have not been applied to SETD6. On the basis of the similarity between the flanking sequences of the lysine-4 and lysine-7 methylation sites within H2AZ, it has been suggested that SETD6 may recognize G.K motifs^58^. In fact, SETD6 was found to promote in vitro methylation of canonical histone proteins at several predicted sites that resemble G.K motifs^58^, such as H4-K12 that was later found independently^57^. Furthermore, this proposed G.K motif is observed in the TWIST1-K33 and E2F1-K117 methylation sites (Fig. 6). However, it is strikingly apparent that the presence of a K.X.V/I/L motif is more common among non-histone substrates of SETD6, especially among physiological non-histone substrates (Fig. 6). In addition to other side-chain interactions between the RELA substrate and SETD6, it is interesting to note that the RELA-I312 residue fits into a surface pocket of SETD6 (ref. ^13^). Given that all the physiological substrates of SETD6 have been identified independently, we anticipate that the apparent K.X.V/I/L motif will be a significant predictor of SETD6 target sites. However, it is important to consider that this apparent motif has been identified from the current literature knowledge and requires experimental validation.

## Biological roles of SETD6

### Hormonal signaling

SETD6 associates with estrogen receptor (ER)α, and this association is enhanced by stimulation with estradiol^86^. In addition, SETD6 associates with several transcriptional coregulators, including HDAC1 and MTA2, which are two subunits of the nucleosome remodeling and deacetylase (NuRD) complex, a repressor of ER-dependent transcription. In agreement with this repressive function, SETD6 confers transcriptional repression of artificial reporters. By contrast, SETD6 also associates with TRRAP, a known coactivator of ER-dependent transcription^86^. Interestingly, SETD6 is a required factor for the expression of endogenous *PGR* and *TFF1*, two estrogen-responsive genes, in certain breast cancer cell lines. However, the expression of *TGFA*, another estrogen-responsive gene, is unaffected by SETD6 silencing. Thus, the role of SETD6 as a regulator of estrogen-responsive genes appears to be contextual regarding cell lines and target genes. In addition to estrogen, a genetic variant of bovine *SETD6* is associated with the conception rate in cattle through the modulation of gonadotropin-releasing hormone^26^. In alignment with the role of SETD6 in suppressing RELA function, bovine SETD6 harboring an SNP that confers increased enzymatic activity was found to repress NF-κB activity and the secretion of gonadotropin-releasing hormone, an NF-κB target gene. In addition, cattle with this SNP in *SETD6* had reduced levels of serum follicle-stimulating hormone.

Overall, SETD6 is involved in the regulation of hormone signaling pathways, and this regulation may be controlled by the enzymatic activity of SETD6 as well as by selective interactions with hormone receptors and chromatin-associated proteins. Hormonal signaling cascades typically involve the induction of PTMs, which, in turn, modulate enzymatic activity and protein‒protein interactions. As mentioned earlier, estradiol enhances the interaction between SETD6 and ERα (ref. ^86^). However, our understanding of cellular PTMs that occur on SETD6, and their functional consequences, remains limited. Deciphering the PTMs that occur on SETD6, particularly those induced by hormonal signaling, is crucial to understanding the role of SETD6 in these pathways.

### Regulation of the NF-κB pathway

Initially, SETD6 was shown to repress NF-κB through RELA-K310 mono-methylation under basal conditions in several cell types^9^. In OSCC, SETD6 represses this pathway by maintaining promoter methylation of *RELA* and *PAK4*, thereby negatively regulating RELA protein levels^22^. Thus, SETD6 may act as a negative regulator of the PAK4 and RELA proteins via both direct protein methylation and indirect regulation of promoter methylation. Whether these pathways occur individually or in concert requires further investigation.

In contrast to its role as a repressor of the NF-κB pathway, SETD6 promotes the activation of this pathway in several cellular contexts. Specifically, SETD6 promotes the nuclear accumulation of p65 in lung adenocarcinoma^20^. In gastric cancer, SETD6 promotes the expression of the p65 protein and the levels of phosphorylated inhibitory kappa B kinase β (ref. ^31^). Finally, in bladder cancer, SETD6 promotes RELA mRNA and protein levels while simultaneously attenuating IκBα mRNA and protein levels in a catalytically dependent manner^21^. SETD6 also promotes the nuclear accumulation of RELA, NF-κB transactivation activity and the expression of several target genes (*CCL2*, *BAX*, *MDM2* and *p21*). SETD6-dependent upregulation of these genes was not observed in the cell types studied by Levy and colleagues^9^, which suggests a cell-type-dependent effect of SETD6 on the NF-κB signaling pathway. Mukherjee and colleagues suggested that contextual differences in cell type confer these opposing responses: (1) methylation recruits activating cofactors that are not present in the U2OS cell line, and (2) the inhibition of IκBα by SETD6 in bladder cancer may be lost in the U2OS cell line^21^.

### Regulation of the Nrf2 pathway

Nuclear factor erythroid 2-related factor 2 (Nrf2) is a master regulator of the antioxidant response to oxidative stress^87^. SETD6 confers differential regulatory control of the Nrf2 pathway depending on the cellular context. Specifically, the silencing or knockout of SETD6 in mouse podocytes and triple-negative human breast carcinoma upregulates Nrf2 and augments the expression of antioxidant Nrf2 target genes^30,88^. SETD6 physically interacts with DJ1, a positive regulator of Nrf2 protein stability and transcriptional response, on chromatin at Nrf2 target genes^30,89^. The SETD6–DJ1 interaction represses Nrf2 activity in response to oxidative stress, and this interaction is lost upon the induction of oxidative stress^30^. In addition to its role as a negative regulator of the Nrf2 pathway, in lung adenocarcinoma, SETD6 promotes the accumulation of Nrf2 in the nucleus, augments the protein expression of Nrf2 and Nrf2 targets and downregulates Keap1, which is a negative regulator of Nrf2 (ref. ^20^). It is likely that differences in the molecular machinery of cell types determine whether SETD6 acts as a promotor or repressor of the Nrf2 pathway.

### Regulation of cancer phenotypes, the cell cycle and apoptosis

Throughout this Review, the role of SETD6 in the regulation of substrate protein function, transcriptional regulation and other biological processes has been discussed in the context of various cancer models. The phenotypic role of SETD6 in each cancer is summarized in Table 3. In particular, SETD6 is known to be a regulator of migration and invasion in glioma, and SETD6 methylation of TWIST1-K33 promotes EMT phenotypes in this cancer type^23^. SETD6 positively influences the migration of glioma, lung adenocarcinoma and gastric cancer cells^20,23,31^. Correspondingly, SETD6 promotes metastatic phenotypes in OSCC^22^. However, in cervical and breast cancer, SETD6 has been shown to repress migration, as well as the invasive properties of the latter^60,90^. SETD6 silencing also suppresses the oxygen consumption rate in breast cancer, and PAK4-K473 methylation attenuates adhesion^60,76^.

**Table 3:** The role of SETD6 in cancer phenotypes.

Cancer type	Phenotypic effects
Breast	SETD6 silencing causes proliferation defects and induces apoptosis^86^. SETD6 enzymatic activity attenuates adhesion, migration and invasion^60^. SETD6 silencing attenuates oxygen consumption rate^76^. Inhibition of SETD6 activity induces proliferation^90^.
Cervical	Inhibition of SETD6 activity induces proliferation and migration^90^. SETD6 knockout enhances cell proliferation^59^.
Lung	SETD6 silencing attenuates migration, colony formation and cell viability^20^.
Bladder	SETD6 positively regulates cell viability, colony formation and cell growth^21^.
Glioma	SETD6 enzymatic activity negatively regulates cell–ECM adhesion and positively regulates migration^23^.
Gastric	SETD6 promotes proliferation, colony formation and migration^31^.
Osteosarcoma	SETD6 activity attenuates cell proliferation^9^.
OSCC	SETD6 silencing attenuates cell proliferation and metastasis, as well as induces apoptosis^22^.

ECM, extracellular matrix.

Silencing of SETD6 causes defects in the proliferation of several breast cancer and OSCC cell lines^86^. In addition, SETD6 drives proliferation in bladder and gastric cancers^21,31^. Conversely, SETD6 activity suppresses the proliferation of breast cancer, cervical cancer and osteosarcoma^9,90^. Nonetheless, SETD6 is positioned as a key regulator of cancer cell proliferation and therefore regulates the cell cycle. Notably, SETD6 methylation of PLK1 slows cervical cancer progression through mitosis and thereby restrains proliferation^59^.

SETD6 and SETD3, which are structurally similar class VII methyltransferases, have common interacting proteins and regulate overlapping transcriptional programs, such as apoptosis-related genes^91^. Functional crosstalk between these two methyltransferases inhibits DNA-damage-induced apoptosis in cervical cancer. Further, SETD6 guards against apoptosis in OSCC and lung adenocarcinoma^20,22^. Therefore, SETD6 promotes cancer cell evasion of apoptosis. By contrast, and outside of a cancer model, SETD6 silencing has been shown to protect mouse podocytes from glucose- and palmitic-acid-induced apoptosis^88^.

### Other biological roles

SETD6 is also involved in hippocampus-dependent memory formation in rats through its regulation of RELA-K310me1 and H3-K9me2, which are learning-induced epigenetic marks^92^. Phenotypically, SETD6 regulates dendritic spine morphology in primary hippocampal neurons, neuronal electrophysiology and long-term memory consolidation.

## Future avenues: therapeutic potential and future research hypotheses

### Current inhibitors and therapeutic potential of SETD6

Historically, peptides have been highly acclaimed as potent and selective mediators of biological processes in vivo. Within the field of lysine methylation, there is a shortage of peptide-based inhibitors that target the enzymatic regulators of methylation and demethylation. Notably, direct inhibition of the SETD6 catalytic pocket has been achieved using a short peptide derived from the RELA-K310 substrate^90^. Because RELA-K310 is modified by SETD6, this short 15-residue peptide (RKRTYETFKSIMKKS) enables precise targeting of the catalytic pocket. Furthermore, when fused to a cell-penetrating peptide (vp22-RELA peptide), this peptide inhibitor binds to SETD6 and blocks SETD6-dependent substrate methylation in vitro and in cells^90^. Treatment of HeLa and MDA-MB-231 cells with the vp22-RELA peptide has been shown to mimic the effect of SETD6 depletion in these cell types by increasing migration and proliferation. Notably, a peptide inhibitor targeting SETD8 methyltransferase was developed by a similar strategy based on the sequence of the histone H4-K20 site, which is a known SETD8 substrate^93^. However, replacing the K20 residue in the H4_16–23_ peptide with norleucine led to the conversion of the natural substrate to a potent in vitro inhibitor. Further, lysine-to-methionine mutations, which are found in oncogenic histones, have also been shown to inhibit the activities of several other methyltransferase^94,95^. Thus, mutations with norleucine, methionine or other natural and non-canonical amino acids may improve the efficiency of the vp22-RELA peptide. Furthermore, peptides derived from other SETD6 substrates (in vitro or physiological substrates) may enable efficient inhibition of SETD6 enzymatic activity.

The potential of natural products to enable potent and selective inhibition is another active area of research. In their study of the role of SETD6 in bladder cancer, Mukherjee and colleagues stated their group has identified palmatine as a potential inhibitor of SETD6 (ref. ^21^). This natural product is isolated from the bark of *Phellodendron amurense*. Mukherjee and colleagues have claimed that this compound decreases the level of SETD6 protein; however, there are currently no data available to corroborate this statement. Palmatine has been shown previously to inhibit growth, invasion and NF-κB activity in prostate cancer cells^96^.

Given the repressive effect that SETD6 has on NF-κB signaling, as demonstrated by Levy and colleagues^9^, there is also therapeutic potential in enhancing SETD6 activity in certain models. For example, overactivation of the NF-κB pathway and cytokine storm are hallmark features of severe coronavirus disease 2019 (COVID-19). Besides the NF-κB activity, SETD6 expression level is downregulated in peripheral blood mononuclear cells from patients with severe COVID-19 and from deceased patients^97^. As a treatment strategy, Kim and colleagues developed a ferritin nanocage decorated with a cell-penetrating and nuclear-localizing peptide to enable the cellular delivery of recombinant SETD6 protein to dampen NF-κB activity. This nanocage structure primarily accumulated in the lungs and kidneys in a mouse model, and it suppressed NF-κB activity and cytokine production in peripheral blood mononuclear cells from patients with severe COVID-19 and rescued the survival rate of septic mouse models that are used for studying cytokine storm^97^. Given that the delivery of recombinant SETD6 protein has therapeutic potential to dampen an overactive immune response, it would be interesting to apply this delivery system to cancer or disease models in which SETD6 antagonizes disease progression.

### Avenues for additional exploration

The methylation‒phosphorylation switch, which governs RELA activity, provided the first evidence that SETD6 methylation participated in crosstalk with other PTMs^9^. Although this has not been studied directly, RELA is likely to be regulated by competition between acetylation and methylation at the K310 residue because these processes have opposing effects on RELA activity^36^. As mentioned, RELA K310 acetylation inhibits neighboring K314/315 methylation^38^. Indeed, methylation of PLK1-K209 and PLK1-K413 inhibit phosphorylation of the adjacent PLK1-T210 residue^59^. The methylation sites on BRD4, E2F1 and PAK4 also overlap or are directly adjacent to other important modifications. Thus, the impact of SETD6 methylation on neighboring PTMs, and vice versa, is another avenue of exploration.

In addition to PTM crosstalk, the site-specific identification or mapping of SETD6 substrates will improve our understanding of SETD6 biology. As mentioned, sequence commonalities among non-histone SETD6 substrates have revealed a K.X.V/I/L motif, which is shared among most substrates in this category (Fig. 6). This motif may be used as a tool to prioritize residues for validation of site-specific methylation from the extensive list of in vitro protein substrates^80^. In addition, we anticipate that SETD6 enzymatic activity may be pertinent to other ribosomal proteins. SETD6 already catalyzes the methylation of MRPS23 and the ribosomal protein RPS27L, and SETD6 interactors are enriched in ribosomal proteins^76,80^.

Finally, it is apparent that SETD6 plays a role in selective transcriptional regulation by acting as a molecular switch at chromatin. This is especially apparent in the ability of SETD6 substrate methylation to dictate the recruitment of additional factors that can direct the genomic localization of the substrate protein, impact their activities at chromatin or alter physical interactions. This is exemplified by BRD4-K99 methylation, which can either block the recruitment of E2F1 to select target genes or enhance BRD4 association with the HPV E2 protein. Identifying the genomic localization and interactomes of methylated and unmethylated SETD6 substrates will pave the way for understanding how SETD6 methylation acts as a regulatory switch that governs selective transcription.

## Conclusion

The lysine methyltransferase SETD6 exerts control over a diverse range of cellular signaling pathways, cellular processes and phenotypes through its enzymatic modification of protein lysine residues. *SETD6* expression is dysregulated in numerous cancers, and this correlates with poor patient survival rates for several types of tumor. Accordingly, SETD6 exerts regulatory control over cancerous phenotypes that are linked to its modification of protein substrates. However, the role of SETD6 as a promotor or suppressor of cancerous phenotypes is contextual regarding cancer type. This contextual regulatory role also extends to several signaling pathways, such as the NF-κB and Nrf2 pathways.

There are several open questions in the SETD6 field that future research should address. (1) What additional factors regulate SETD6? (2) What are the other physiological substrates of SETD6, and what are the downstream biological effects of their methylation? (3) Do any lysine demethylases counteract SETD6 substrate methylation? (4) What cellular factors dictate whether SETD6 plays a promotor or suppressor role over specific signaling pathways and cancerous phenotypes? (5) What is the role of SETD6 in the cytoplasm? (6) What other site-specific PTMs does SETD6 substrate methylation have functional crosstalk with? (7) What is the role of SETD6 in chromatin and selective transcriptional regulation? The current landscape of physiological and in vitro SETD6 substrates in combination with the future application of omics approaches will address these gaps and improve our understanding of the role of SETD6 in human health and disease.
